# Predictive risk score of respiratory complications in children with mediastinal tumors: A case–control study

**DOI:** 10.1002/cam4.4972

**Published:** 2022-06-23

**Authors:** Mamoru Honda, Arakawa Yuki, Hosokawa Takahiro, Oyama Chigusa, Mitani Yuichi, Mori Makiko, Kohei Fukuoka, Oshima Koichi, Tanami Yutaka, Ishimaru Tetsuya, Kawashima Hiroshi, Mizuta Koichi, Ueta Ikuya, Kuratani Norifumi, Koh Katsuyoshi

**Affiliations:** ^1^ Department of Hematology/Oncology Saitama Children's Medical Center Saitama Japan; ^2^ Department of Radiology Saitama Children's Medical Center Saitama Japan; ^3^ Department of Pediatric Surgery Saitama Children's Medical Center Saitama Japan; ^4^ Department of Pediatric Intensive Care Saitama Children's Medical Center Saitama Japan; ^5^ Department of Anesthesia Saitama Children's Medical Center Saitama Japan

**Keywords:** children, mediastinal tumor, respiratory complications, risk score, tracheal cross‐sectional area

## Abstract

**Background:**

The aim of this study was to examine risk factors of respiratory complications at the diagnosis and establish an algorithm of clinical management in children and adolescents with mediastinal tumors.

**Methods:**

We retrospectively collected clinical information of all children and adolescents who presented with mediastinal tumors at Saitama Children's Medical Center from 1999 to 2019, including age, sex, pathological diagnosis, eight major clinical symptoms (cough, dyspnea, hypoxia, orthopnea, chest pain, wheeze, superior vena cava syndrome, and stridor), chest computed tomography (CT) findings (tumor location, mediastinal mass ratio, pleural fluid, pericardial effusion, and compression of trachea and bronchi), types of diagnostic procedure and anesthesia, respiratory complications (severe hypoxia, difficult ventilation, respiratory failure, and cardiopulmonary arrest), and clinical outcome. Subsequently, we calculated the risk score for predicting respiratory complications by combining clinical and radiological findings.

**Results:**

Of the 57 patients, 7 (12%) developed respiratory complications. Cough, dyspnea, hypoxia, and orthopnea were significantly more common in patients with complications (*p* = 0.02, *p* = 0.02, *p* < 0.01, *p* = 0.03, respectively). The reduction of percentage of tracheal cross‐sectional area (%TCA) and compression of the carina in chest CT were also significantly more common in patients with complications (*p* < 0.01 and <0.01, respectively). We calculated the risk score of respiratory complications by combining cough, wheeze, stridor, orthopnea, dyspnea, hypoxia, %TCA < 0.5, and compression of the carina. A risk score ≥ 7 showed high predictive accuracy for complications (sensitivity: 100%, specificity: 97.7%, positive likelihood ratio: 43.0).

**Conclusion:**

The risk score combining clinical symptoms with radiological findings is a promising predictive tool for respiratory complications in children with mediastinal tumors.

## Graphical abstract

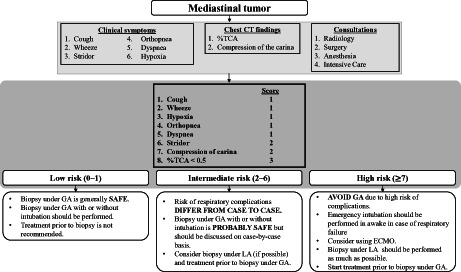

## 1 INTRODUCTION

Children with mediastinal tumors are at risk of respiratory complications due to airway obstruction at the onset of disease.^1^, ^2^, ^3^, ^4^, ^5^, ^6^, ^7^ Diagnosis and treatment in mediastinal tumors are associated with conflicting therapeutic goals, whereby an attempt to treat (corticosteroids or chemotherapy in malignancy) or diagnose (biopsy, thoracentesis, pericardiocentesis) can either alter the diagnosis or de‐stabilize the patient. Thus, identifying patients who are at risk of respiratory complications is important.

Several clinical symptoms have been reported as risk factors for respiratory complications: stridor, orthopnea, wheeze, and ≥3 clinical symptoms.^3^, ^4^, ^5^, ^6^ In addition, radiological findings (e.g., compression of great vessels, trachea, carina, and bronchi) have been reported as useful findings for predicting airway complications.^2^, ^4^, ^5^ Specifically, reduction in the % of tracheal cross‐sectional area (%TCA) has been reported as a useful risk factor of respiratory complications.^2^, ^5^ However, the usefulness of each risk factor differs among studies, suggesting that there is no definitive single risk factor for precisely predicting respiratory complications.

However, there are limited data regarding the accuracy of each risk factor, and the usefulness of these risk factors in clinical management, such as determining the anesthetic technique and diagnostic procedure or initiating treatment prior to performing the diagnostic procedures. In this study, we analyzed a cohort of patients with mediastinal tumors in a single institute over the past 20 years to retrospectively examine the accuracy of risk factors of respiratory complications, develop a predictive risk score and establish an algorithm of clinical management, including the determination of the anesthetic technique, diagnostic procedure, and treatment prior to performing the diagnostic procedures.

## 2 METHODS

### 2.1 Patient data

Clinical records of all children and adolescents who presented with mediastinal tumors to Saitama Children's Medical Center from 1999 to 2019 were retrospectively analyzed. Patients aged 1–18 years who were diagnosed with mediastinal masses by radiologists in our institute were enrolled in this study. Clinical information, including age, sex, pathological diagnosis, eight major clinical symptoms (i.e., cough, dyspnea, hypoxia, orthopnea, chest pain, wheeze, SVC syndrome, and stridor), chest computed tomography (CT) findings, types of diagnostic procedure and anesthesia, complications and clinical outcome were collected from the clinical records. Types of anesthesia were categorized as GA (using sedatives and analgesics without intubation), GA+ (GA with intubation), and local anesthesia (LA).

### 2.2 General approach to patients with mediastinal mass

In all patients with mediastinal mass detected by chest X‐ray, laboratory tests including tumor markers (serum soluble IL‐2 receptor, α‐fetoprotein, human chorionic gonadotropin, neuron‐specific enolase, and urinary vanillylmandelic acid and homovanillic acid) were checked. The cervical to thoracoabdominal CT scan with contrast was generally performed in supine position. In cases with orthopnea, the CT scan was performed in half‐lying position or Fowler's position. For the pathological diagnosis, less invasive tests, such as bone marrow aspiration (BMA) and thoracentesis, were preferred. Mediastinal tumor biopsy was performed if diagnosis was not obtained from these less invasive tests. Type of anesthesia (LA, GA, and GA+) was selected according to diagnostic procedure and risk of respiratory complications. In patients who were assessed very high risk at respiratory complications in diagnostic procedures, administration of corticosteroids or chemotherapy prior to diagnostic procedures were started.

### 2.3 Radiological assessments

Chest CT findings (tumor location, %TCA, mediastinal mass ratio [MMR], pleural effusion, compression of vessels, compression of bronchi and carina, and pericardial effusion) were assessed by a radiologist at our institute. Then, the percentage of the findings directly related to respiratory complications (pleural fluid, pericardial effusion, compression of the carina, compression of the left and right bronchi, %TCA, and MMR) was calculated.

**FIGURE 1:**
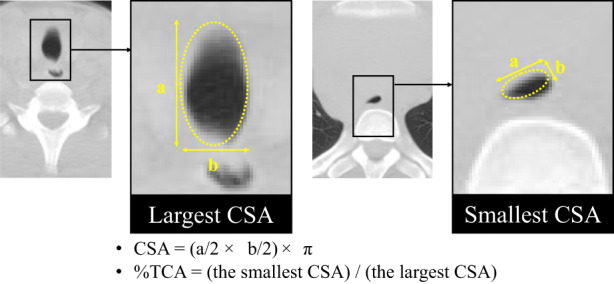
Calculation of %TCA. %TCA is calculated by the ratio of the smallest CSA to the largest CSA. The CSA is approximated by the formula of an eclipse. CSA, cross‐sectional area; %TCA, % of tracheal cross‐sectional area; a, widest diameter of lumina; b, narrowest diameter of lumina.

Compression of the bronchi and carina and %TCA were evaluated using axial CT image (window level: −400 and window width: 1600) on a 1600 × 1200 picture archiving and communication system monitor (PACS; GE Healthcare). The %TCA was calculated by the cross‐sectional area (CSA) of a dimension of the trachea at its smallest and largest lumina using the following formula of an ellipse (Figure 1):

CSA = (widest diameter/2 × narrowest diameter/2) × π.

%TCA = (the smallest CSA)/(the largest CSA).

Pleural effusion, compression of vessels, and pericardial effusion were evaluated using enhanced CT images. MMR was defined as the maximum width of the mediastinal mass divided by the maximum intrathoracic width on the CT image, which was typically measured at the level of the diaphragm.

### 2.4 Respiratory complications

Respiratory complications were defined as at least one of the following events observed from admission to biopsy: severe hypoxia (oxygen saturation [SpO_2_] < 70% at room air); difficult ventilation (events the patients were ventilated by mask bagging during general anesthesia because of obstructive apnea or hypoxia); respiratory failure (requiring emergency intubation); and cardiopulmonary arrest (CPA).

### 2.5 Accuracy of risk factors and risk scoring for predicting respiratory complications

Risk factors were determined based on our study results and previous studies.^2^, ^4^, ^5^ The accuracy of these risk factors for predicting the occurrence of respiratory complications was evaluated according to the sensitivity, specificity, positive predictive value, negative predictive value, odds ratio, and likelihood ratio (LR). Each score of these risk factors ranged from 1 to 3 points, which depended on the value of positive LR, and the risk scoring of maximum positive LR was adopted.

### 2.6 Statistical analysis

Associations of patient characteristics (e.g., clinical symptoms, CT findings, and pathological diagnosis) with respiratory complications were analyzed using Fisher's exact test and the Mann–Whitney *U* test. *p* values ≤0.05 denoted statistical significance. Receiver Operatorating Characteristic (ROC) curves are drawn from the points plotted based on sensitivity and specificity at each risk score. The risk score with the highest area under the curve (AUC) was calculated as the cutoff value. All statistical analyses were performed with EZR software.^8^

### 2.7 Ethics

This study was approved by the ethics committees of the Saitama Children's Medical Center (No. 2021–02‐001).

## 3 RESULTS

### 3.1 Patient characteristics

The characteristics of patients included in this study are shown in Table 1. Between 1999 and 2019, 57 patients (36 males; 21 females) who presented with mediastinal tumors were admitted to our hospital. The median age at diagnosis was 10.2 (range: 1.7–15.7) years. Histological diagnosis was established in 54 patients: T‐cell lymphoblastic lymphoma (T‐LBL) (*n* = 25); T‐cell acute lymphoblastic leukemia (*n* = 9); Hodgkin lymphoma (*n* = 7); mature teratoma (*n* = 4); Anaplastic large cell lymphoma (ALCL) (*n* = 3); B‐cell lymphoblastic lymphoma (B‐LBL) (*n* = 1); Primary mediastinal B‐cell lymphoma (PMBCL) (*n* = 1); Ewing sarcoma (*n* = 1); thymolipoma (*n* = 1); lipoma (*n* = 1); thymic cyst (*n* = 1); and acute myeloid leukemia (*n* = 1). One patient was diagnosed with a malignant germ cell tumor by elevated serum levels of human chorionic gonadotropin‐β and alpha fetal protein. The other two patients were diagnosed with T‐LBL because the tumors were originated from thymus. Respiratory symptoms were common in patients with mediastinal tumors: cough (*n* = 24); dyspnea (*n* = 17); hypoxia (*n* = 17); orthopnea (*n* = 12); wheeze (*n* = 8); and stridor (*n* = 7). Chest pain and SVC syndrome were also observed (*n* = 10 and 8, respectively).

**TABLE 1:** Patient characteristics

	Total	Complications (+)	Complications (−)	*p*‐value
Number	57	7 (12%)	50 (88%)	
Median age at diagnosis, years	10.2	11.7	8.9	0.75
Sex				0.49
Male	36 (63%)	5	31	
Female	21 (37%)	3	18	
Symptoms				
Cough	24 (42%)	6 (86%)	18 (36%)	0.02
Hypoxia (SpO_2_: <95%)	18 (32%)	6 (86%)	12 (24%)	<0.01
Dyspnea	17 (30%)	5 (71%)	12 (24%)	0.02
Orthopnea	12 (21%)	4 (57%)	8 (16%)	0.03
Chest pain	10 (18%)	0	10 (20%)	0.24
Wheeze	8 (14%)	3 (43%)	5 (10%)	0.99
Superior vena cava syndrome	8 (14%)	0	8 (16%)	0.32
Stridor	7 (12%)	3 (43%)	4 (8%)	1
Number of symptoms (median)	1.0	3.0	0.5	<0.01
Chest CT findings (*n* = 52)				
Pleural fluid	19 (33%)	1 (14%)	18 (36%)	0.19
Pericardial effusion	16 (28%)	4 (57%)	12 (24%)	0.12
Compression of the carina	13 (23%)	6 (86%)	7 (14%)	<0.01
Compression of left bronchi	11 (19%)	2 (29%)	9 (18%)	0.85
Compression of right bronchi	4 (7%)	0	4 (8%)	0.55
% of tracheal cross‐sectional area (SD)	0.83 (0.23)	0.39 (0.11)	0.84 (0.20)	<0.01
Mediastinal mass ratio (SD)	0.40 (0.15)	0.52 (0.08)	0.39 (0.16)	0.16
Diagnosis				0.02
T‐lymphoblastic lymphoma	25	6	19	
B‐lymphoblastic lymphoma	1	0	1	
Anaplastic large cell lymphoma	3	0	3	
Primary mediastinal B‐cell lymphoma	1	1	0	
Hodgkin lymphoma	7	0	7	
T‐cell acute lymphoblastic leukemia	9	0	9	
Burkitt leukemia	1	0	1	
Acute myeloid leukemia	1	0	1	
Teratoma	4	0	4	
Malignant germ cell tumor	1	0	1	
Lipoma/thymolipoma	2	0	2	
Thymic cyst	1	0	1	
Ewing sarcoma	1	0	1	

Abbreviations: CT, computed tomography; SD, standard deviation.

### 3.2 Chest CT findings

The characteristics of the chest CT images of 52 patients are shown in Table 1. For the remaining five patients, a chest CT image on admission was not obtained. The locations of mediastinal tumors varied: anterior (*n* = 12); anterior‐middle (*n* = 30); anterior‐middle‐posterior (*n* = 7); middle (*n* = 1); and chest wall (*n* = 2). Tumors located on the anterior chest wall were lipomas and B‐LBL. All tumors, which caused complications, were located in the anterior or anterior‐middle mediastinum.

### 3.3 Respiratory complications

In our study, 7/57 patients (12%) developed respiratory complications. Cough, dyspnea, hypoxia, and orthopnea were significantly more common in patients with complications (*p* = 0.02, *p* = 0.02, *p* < 0.01, *p* = 0.03, respectively). The median number of the eight major clinical symptoms was 3.0 and 0.5 in patients with and without complications, respectively (Table 1). None of the patients with 0–1 clinical symptom developed respiratory complications. Regarding chest CT findings, compression of the carina was significantly more frequent in patients with complications (*p* < 0.01), and the %TCA in patients with complications (0.39) was significantly lower than the patients without complications (0.84) (*p* < 0.01). Other findings (e.g., pleural fluid, pericardial effusion, compression of bronchi, and MMR) were not significantly different between the two groups (Table 1).

Table 2 shows the details of seven patients with respiratory complications. Respiratory complications occurred on admission (Cases 3 and 4), prior to GA with intubation (Case 5), during GA (Case 2), during GA with intubation (case 6 and 7), and after awake intubation (Case 1). Case 1 was admitted to our hospital with severe respiratory distress. GA was not performed because the patient was considered at high risk of airway obstruction; thus, he was intubated in the awake state. However, he developed difficult ventilation due to airway obstruction and expired due to complete airway obstruction 3 days after admission. Cases 3 and 4 were admitted to our hospital with respiratory failure and required emergency intubation. They recovered after emergency intubation, received chemotherapy, and are currently alive without relapse. Case 5 developed difficult ventilation in the supine position before GA. Cases 2 and 6 developed respiratory complications during the diagnostic procedure under GA but recovered after bagging or changing the body position from the supine position to the prone. Case 7 underwent cervical lymph node biopsy under GA with intubation in the supine position, but experienced CPA due to airway obstruction during the diagnostic procedure. He recovered after cardiopulmonary resuscitation and was extubated after receiving chemotherapy. However, he suddenly expired due to massive hemoptysis caused by tracheal bleeding 18 days after admission. Pathological autopsy revealed bleeding from the tracheoesophageal fistula.

**TABLE 2:** Details of patients with respiratory complications

Case No.	Age Sex	Diagnosis	Clinical symptoms	%TCA	CT findings	Diagnostic procedure	Risk score	Details of complications	Outcome
1	4.5 M	T‐LBL^b^	Cough, wheeze, stridor, orthopnea, dyspnea, hypoxia	N.A (intubated)	Compression of left bronchus and carina, pericardial effusion	Needle biopsy of mediastinal tumor under LA	N.A^a^ (≥9)	Difficult ventilation after awake intubation for needle biopsy of mediastinal tumor	Death due to complete airway obstruction (3 days after diagnosis)
2	9.1 M	T‐LBL	Cough, orthopnea, dyspnea, hypoxia	0.39	Pleual effusion, compression of carina, pericardial effusion	Thoracocentesis under GA without intubation	9	Difficult ventilation and transient severe hypoxia (SpO_2_: 48%) during GA for thoracocentesis	Recovery after mask bagging and changing to sitting position
3	6.7 M	T‐LBL^b^	Cough, wheeze, hypoxia, orthopnea, dyspnea	N.A (intubated)	Compression of carina	BMA and biopsy of mediastinal tumor under GA with intubation	N.A^a^ (≥7)	Respiratory failure on admission	Recovery after emergency intubation
4	12.7 M	T‐LBL	Stridor, orthopnea, dyspnea	N.A (intubated)	None	Cervical lymph node biopsy under GA with intubation	N.A^a^ (≥4)	Respiratory failure on admission	Recovery after emergency intubation
5	11.7 F	T‐LBL	Cough, stridor, dyspnea	0.38	Compression of carina	BMA under LA and biopsy of mediastinal tumor under GA with intubation	9	Difficult ventilation and severe hypoxia (SpO_2_: 40%) in the supine position	Recovery after mask bagging
6	14.9 F	PMBCL	Cough, wheeze, hypoxia	0.47	Compression of left bronchus and carina, pericardial effusion	Cervical lymph node biopsy under GA with intubation	8	Transient severe hypoxia and bradycardia due to difficult ventilation during GA for cervical lymph node biopsy	Recovery after mask bagging
7	14.6 M	T‐LBL	Cough, hypoxia	0.18	Compression of carina, pericardial effusion	Cervical lymph node biopsy under GA with intubation	7	Difficult ventilation, cardiopulmonary arrest during GA	Recovery after CPR, sudden death due to tracheal bleeding (18 days after diagnosis)

Abbreviations: BMA, bone marrow aspiration; CPR, cardiopulmonary resuscitation; CT, computed tomography; F, female; GA, general anesthesia; LA, local anesthesia; M, male; N.A, not available; PMBCL, primary mediastinal large B‐cell lymphoma; T‐LBL, T‐cell lymphoblastic lymphoma; %TCA, % of tracheal cross‐sectional area.

^a^ Cases 1, 3, and 4 required emergency intubation before undergoing CT scanning; risk scores of the three patients were not available because %TCA were not calculated due to tracheal tube.

^b^ Histological diagnosis was not obtained because of the inadequate tumor sample.

### 3.4 Accuracy of clinical and radiological risk factors for predicting respiratory complications

We evaluated the accuracy of major clinical and radiological findings for predicting respiratory complications: cough; wheeze; stridor; orthopnea; dyspnea; hypoxia; %TCA; and compression of the carina (Table 3). Positive LR was highest in the %TCA (7.3) followed by compression of the carina (5.4), stridor (5.4), wheeze (4.3), orthopnea (3.6), hypoxia (3.6), dyspnea (3.0), and cough (2.4).

**TABLE 3:** Accuracy of clinical and radiological risk factors for predicting respiratory complications

	Sensitivity (%)	Specificity (%)	Odds ratio	Positive LR	Negative LR
Cough	85.7	64.0	10.7	2.4	0.2
Wheeze	42.9	90.0	6.8	4.3	0.6
Stridor	42.9	92.0	8.6	5.4	0.6
Orthopnea	57.1	84.0	7.0	3.6	0.5
Dyspnea	71.4	76.0	7.9	3.0	0.4
Hypoxia	85.7	76.0	19.0	3.6	0.2
%TCA < 0.5	100.0	86.4	∞	7.3	0.0
Compression of carina	85.7	84.1	31.7	5.4	0.2
**Risk score** ^**a**^ **≥** **7**	**100.0**	**97.7**	**∞**	**43.0**	**0.0**

Abbreviations: LR, likelihood ratio; %TCA, % of tracheal cross‐sectional area.

^a^ Total score of the following: cough (1), wheeze (1), stridor (2), orthopnea (1), dyspnea (1), hypoxia (1), %TCA < 0.5 (3), compression of carina: (2).

We calculated the risk score for predicting respiratory complications by combining clinical and radiological findings (Table 3) in 47 patients. In the remaining 10 patients, five patients were intubated before undergoing chest CT and we could not obtain %TCA. The other five patients were lack of chest CT data on admission. This risk score consists of eight major clinical and radiological findings. %TCA < 0.5 had the highest positive LR of all risk factors (3 points). Stridor and compression of the carina had the second highest positive LR (2 points, respectively). Cough, wheeze, orthopnea, dyspnea, and hypoxia had relatively low positive LR, but were all important symptoms suggesting respiratory distress (1 point, respectively). As a result, the ROC curve analysis showed that a total score of ≥7 had the highest AUC (0.977) (Figure S1). Risk scores ≥7 had the highest sensitivity (100%), specificity (97.7%), odds ratio (infinity), and positive LR (43.5) among all other single risk factors. The risk score was significantly higher in patients who received local anesthesia (LA) than GA (median score: 5 and 0, respectively) (*p* < 0.01).

### 3.5 Diagnostic approach and clinical outcome by the risk score

**FIGURE 2:**
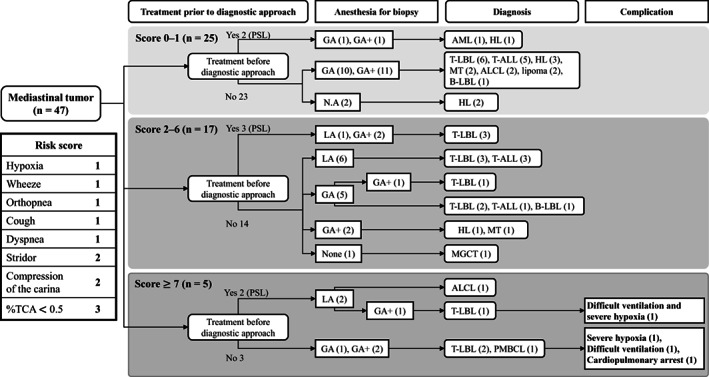
A retrospectively validated diagnostic approach and outcome based on the risk score. Children with mediastinal tumors (*n* = 47) were divided into three groups according to their risk score: score 0–1 (*n* = 25); 2–6 (*n* = 17); and ≥7 (*n* = 5). Respiratory complications occurred only in patients with a risk score ≥ 7. AML, acute myeloid leukemia; ALCL, anaplastic large cell lymphoma; BL, Burkitt lymphoma; B‐LBL, B‐cell lymphoblastic lymphoma; GA, general anesthesia; GA+, general anesthesia with intubation; HL, Hodgkin lymphoma; LA, local anesthesia; MGCT, malignant germ cell tumor; MT, mature teratoma; PMBCL, primary mediastinal B‐cell lymphoma; PSL, prednisolone; T‐ALL, T‐cell acute lymphoblastic leukemia; T‐LBL, T‐cell lymphoblastic lymphoma.

We retrospectively compared the diagnostic approach and clinical outcome of patients according to the risk score. In all patients with a risk score of 0–1 (*n* = 25), pretreatment diagnostic procedures were performed under GA without complications in 21/23 patients. In patients with a total score of 2–6 (*n* = 17), the diagnostic procedures were performed under LA or GA depending on the patients' age, as well as the conditions and site for biopsy, without complications. Seven of the 14 patients who had not received treatment prior to the diagnostic procedures all safely underwent GA. Among those with a total score of ≥7 (*n* = 5), four patients developed respiratory complications. Of those, three patients developed respiratory complications during the diagnostic procedure under GA without administration of prednisolone prior to the diagnostic procedures (Figure 2).

## 4 DISCUSSION

In this study, we evaluated clinical and radiological findings as factors for predicting the risk of respiratory complications, calculated the accuracy of risk factors, and established a risk scoring method for proper management. Our study showed that a certain proportion of patients developed respiratory complications. Cough, wheeze, hypoxia, %TCA, and compression of the carina were significant clinical and radiological signs in patients with respiratory complications. This analysis did not reveal a single strong risk factor adequate for predicting complications. However, risk score produced by combining eight major clinical and radiological findings was the most useful and strongest risk factor.

Respiratory complications in patients with mediastinal tumors are usually observed during GA, and the incidence of complications ranges 9.4%–19.6%.^1^, ^2^, ^3^, ^4^, ^5^, ^6^, ^7^ Ng et al. reported anesthetic outcome and predictive risk factors in 48 children with mediastinal tumors who underwent GA; of those, seven children (15%) developed complications during GA.^4^ Anghelescu et al. reported that 11 of 117 children (9%) with mediastinal tumors developed complications during GA.^3^ However, complications can be occasionally observed in other situations: during transport to the operating room for pericardial drainage procedure,^9^ and when assuming the supine position for phlebotomy and CT scanning.^6^ In this study, one of seven children with complications developed severe hypoxia due to airway obstruction after assuming the supine position prior to GA. The supine position can induce the occurrence of respiratory complications regardless of the anesthetic procedure employed.

**FIGURE 3:**
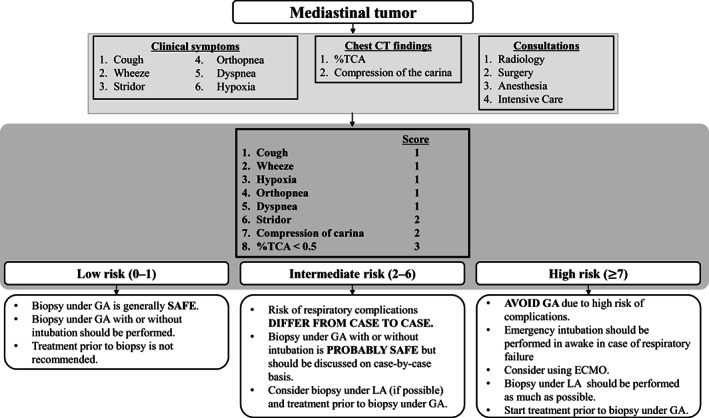
Algorithm for the workup of patients with mediastinal tumors. In patients with mediastinal tumors, we evaluated clinical symptoms and chest CT findings, and consulted with radiologists, surgeons, anesthesiologists, and intensivists. Next, risk stratification is performed by risk scoring. Clinical management was determined as per risk groups. CT, computed tomography; GA, general anesthesia; LA, local anesthesia; %TCA, % of tracheal cross‐sectional area, ECMO, extracorporeal membrane oxygenation.

Several risk factors for predicting complications have been reported previously: stridor, orthopnea, SVC syndrome, ≥3 clinical symptoms, compression of great vessels, trachea, carina, bronchi, and reduction of the %TCA.^2^, ^3^, ^4^, ^5^, ^6^ However, there is limited data about how these risk factors are useful for accurately predicting the occurrence of complications. Ng et al.^4^ were the only ones who have reported the accuracy of risk factors for risk of complications in patients with mediastinal tumors. They reported the accuracy of several risk factors: diagnosis of NHL (sensitivity 85.7%, specificity 80.5%), mediastinal location (85.7%, 58.5%), tumor bulk (85.7%, 58.5%), ≥3 respiratory symptoms (100%, 82.9%), tracheal compression (85.7%, 100%), vascular compression (85.7%, 82.9%), infection (71.4%, 85.4%), and type of procedure (85.7%, 17.1%). Tracheal compression and the presence of ≥3 respiratory signs and symptoms were reported to be the strongest predictive factors, but the accuracy was not adequate for precisely predicting respiratory complications. Furthermore, there are limited data regarding the usefulness of these risk factors in clinical management, such as determining the anesthetic technique and diagnostic procedure or initiating treatment prior to performing the diagnostic procedures. In this study, cough, dyspnea, hypoxia, orthopnea, the number of clinical symptoms, compression of the carina, and the %TCA were significant in patients with complications. However, the accuracy of these factors for predicting complications was not satisfactory. Therefore, we derived a risk score for predicting respiratory complications by combining clinical symptoms (i.e., cough, wheeze, stridor, orthopnea, dyspnea, and hypoxia) and radiological findings (%TCA < 0.5 and compression of the carina). As a result, this risk score showed high accuracy and was highly predictive of respiratory complications. We also produced an algorithm for the workup of patients with mediastinal tumors using the risk score (Figure 3). A low risk score (0–1) indicates that the risk of complications is low and biopsy under GA is generally safe. An intermediate risk score (2–6) suggests that the risk of complications is generally low, but biopsy under LA should be selected if possible. A high risk score (≥7) indicates that biopsy under GA should be avoided. Moreover, intubation to the narrow lumen of the trachea might have a risk of tracheal injury like Case 7 on Table 2. Thus, emergency treatment prior to diagnostic procedure under GA must be considered.

The usefulness of non‐Hodgkin lymphoma (NHL) diagnosis as a predictor of respiratory complications is controversial. King et al. reported that pulmonary function is significantly decreased in patients with NHL.^9^ Nevertheless, Ng et al. reported that ≥50% of NHL cases did not have complications; hence, NHL diagnosis is not a strong predictive factor for respiratory complications.^4^ In the present study, all patients with complications were diagnosed with NHL (T‐LBL 6, PMBCL 1). However, most of NHL cases (23/30, 77%) did not develop respiratory complications. Therefore, the results of the present study also confirm those of previous investigations showing that a diagnosis of NHL is not a strong predictive factor for respiratory complications.

There were some limitations in this study. First, this was a retrospective analysis carried out in patients at a single institute; hence, there were some biases in this study, which influence respiratory complications, such as the selection of anesthetic technique (GA or LA), patients' body position, and emergency treatment with prednisolone prior to performing the diagnostic procedure. Second, the risk score was not calculated for 10 patients due to the lack of CT images or emergency intubation on admission. Although these patients were at high risk of complications, their risk score was not evaluated. These biases can reduce the accuracy of the risk score. The usefulness of this risk score should be prospectively examined. From 2021, we have prospectively validated risk score in four patients with mediastinal tumor. One patient with low risk (score 0) was diagnosed with T‐ALL by BMA under GA. There were three cases with intermediate risk (score 2, 5, and 6, respectively). One patient (score 2) was diagnosed with T‐LBL by mediastinal tumor biopsy under GA+. The remaining two patients (score 5 and 6) were diagnosed with T‐ALL by BMA under GA, and by BMA and thoracentesis under LA. All 4 patients underwent diagnostic procedures without complications, which suggests the usefulness of this risks core.

Our study shows that evaluating both clinical symptoms and the chest CT images is important to accurately determine the risk of respiratory complications, with the exception of patients with severe respiratory failure on admission who require emergency intubation. There is no single risk factor adequate for precisely predicting respiratory complications. We developed a novel risk score combining clinical symptoms with radiological findings. The risk score is a promising tool for predicting the occurrence of respiratory complications and indicating the appropriate clinical management including diagnostic procedures and treatment prior to biopsy in children with mediastinal tumors.

## AUTHOR CONTRIBUTIONS

Mamoru Honda: Conceptualization, data curation, formal analysis, and writing–original draft. Yuki Arakawa: Conceptualization, methodology, and supervision. Takahiro Hosokawa: Data curation, formal analysis, and writing–reviewing and editing. Chigusa Oyama: Data curation, investigation, and writing–reviewing and editing. Yuichi Mitani, Makiko Mori, Kohei Fukuoka, Koichi Oshima, Yutaka Tanami, Tetsuya Ishimaru, Hiroshi Kawashima, Koichi Mizuta, Ikuya Ueta, and Norifumi Kuratani: Writing–reviewing and editing. Katsuyoshi Koh: Supervision, project administration, and Writing–reviewing and editing.

## FUNDING INFORMATION

No specific funding was disclosed.

## CONFLICT OF INTEREST

None.

## Supporting information


Figure S1

## Data Availability

The data that support the findings of this study are available on request from the corresponding author.
