# Supplementary figures and images for: Predictive risk score of respiratory complications in children with mediastinal tumors: A case–control study
Source: Cancer Med. 2022 Jun 23;12(2):1167–76. doi: 10.1002/cam4.4972 (PMC9883441; doi:10.1002/cam4.4972)

Supplemental Fig. 1

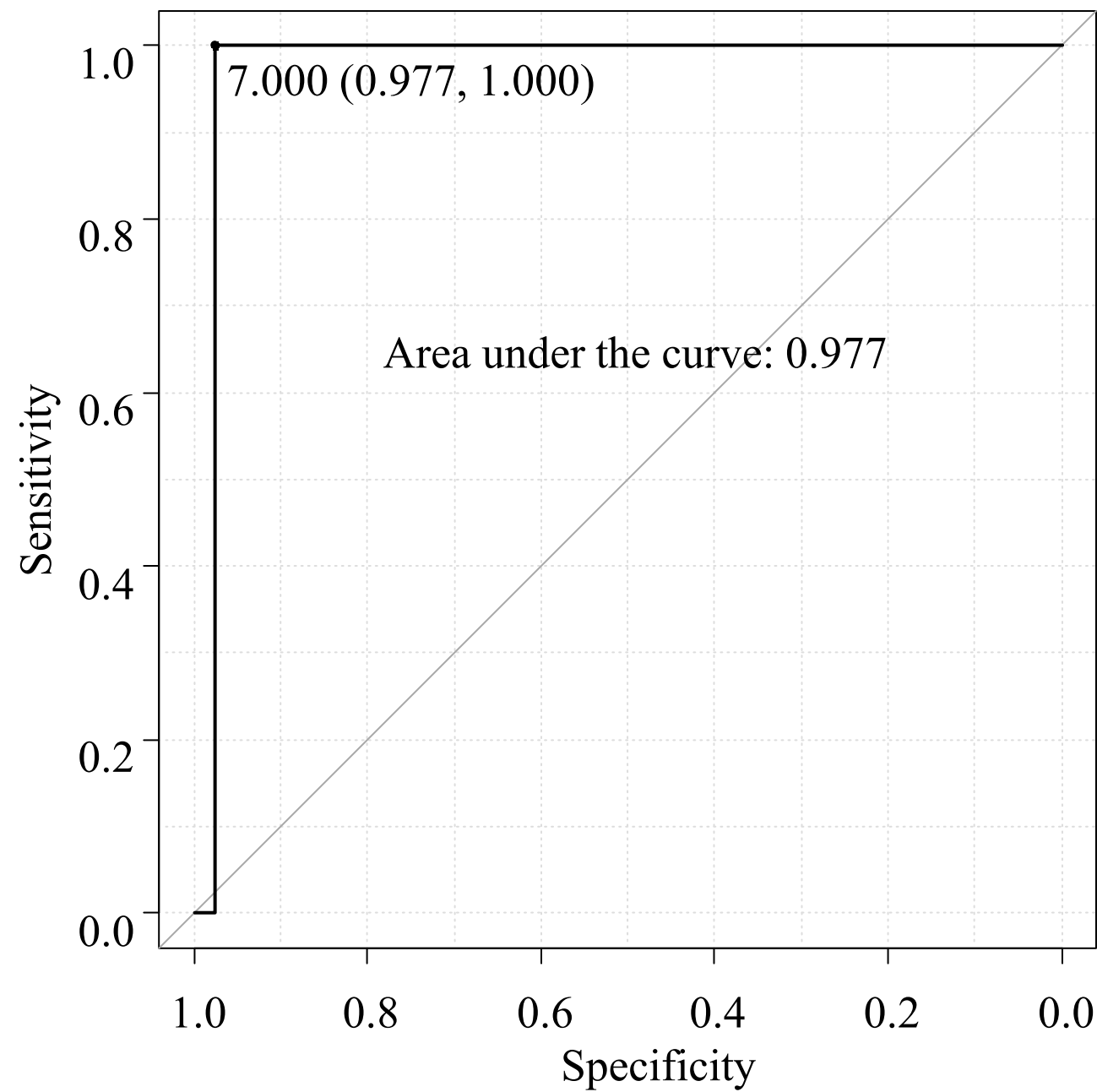

Supplement: Supplementary file 1 — Figure S1 [file CAM4-12-1167-s001.pdf]
